# Primary care-based surveillance to estimate the proportion of rotavirus gastroenteritis among Latvian children below 5 years of age with acute gastroenteritis

**DOI:** 10.1080/21645515.2018.1534515

**Published:** 2018-10-31

**Authors:** Monica Tafalla, Dace Gardovska, Kusuma Gopala, Liga Kozlovska

**Affiliations:** a Epidemiology & Health Outcomes, GSK, Wavre, Belgium; b Paediatric Department, Riga Stradiņš University, Children’s University Hospital, Riga, Latvia; c GSK, Bangalore, India; d Biostatistics EPI, Riga Stradiņš University, Riga, Latvia

**Keywords:** acute gastroenteritis, Latvia, rotavirus, RV disease burden, primary care settings

## Abstract

**Background**: Rotavirus (RV) is worldwide an important cause of acute gastroenteritis (AGE) in infants and young children. There is no specific treatment for AGE caused by RV (RVGE) but since 2006 two safe and effective vaccines have been available. RV vaccination was included in the national immunization program (NIP) of Latvia in 2015 with full reimbursement, and within the first year a coverage of 87% was achieved. This surveillance study was carried out to investigate the proportion of RVGE among AGE episodes in Latvia up to the inclusion of RV vaccination in the NIP to provide a basis for future assessments of the impact of RV vaccination.

**Methods**: Prospective, one-year observational study of children younger than 5 years presenting with AGE in the primary care setting. At first primary care contact, a stool sample was collected and tested for RV using a rapid, visual immunochromatographic kit. The parents monitored their child’s symptoms over 2 weeks after the first contact and the investigator recorded these observations during a follow-up phone call. The proportion of RVGE among the AGE cases was estimated and the severity of each AGE case was assessed based on the recorded symptoms using the 20-point Vesikari scale. The seasonality of RVGE was also investigated.

**Results**: Fifty-two primary care investigators collected data on 606 evaluable children with AGE. The proportion of RVGE was 38.1%. Severe AGE was experienced by 40.7% of the RV-positive and 19.5% of the RV-negative patients. The rate of hospitalization was 9.1% for the RV-positive and 4.8% for the RV-negative with no difference in the mean duration of hospital stays. AGE and RVGE both occurred all year round but with a clearly marked peak only for RVGE, from March to May.

**Conclusion**: This study underlines that RV is an important cause of AGE in children under 5 years old in Latvia and that the burden of disease of RVGE in primary care was substantial before inclusion of RV vaccination in the NIP.

**Trial registration**: NCT01733849

## Introduction

Acute rotavirus gastroenteritis (RVGE) is a universal disease, although the etiological role of rotavirus (RV) is often unrecognized as it requires confirmation by laboratory test.^1–3^ RV is transmitted primarily via the fecal-oral route and it attacks and destroys the enterocytes of the intestinal villi, thereby diminishing their absorptive capacity and causing diarrhea.^1^ Transmission occurs either directly from person-to-person or indirectly via contaminated surfaces, where the RV may persist for extended periods and be transmitted to susceptible individuals.^4^

Clinically, the disease can vary from subclinical, asymptomatic forms, which are more common in older children and adults, to acute gastroenteritis (AGE) with vomiting, watery diarrhea and fever.^5^ In some cases, the disease can progress with severe diarrhea accompanied by vomiting and risk of dehydration, which may rapidly become irreversible and fatal if not corrected adequately from the beginning.^3,5^ The progression to life-threatening dehydration is unpredictable, as there are no recognized risk factors for such progression.^3^

Newborns are partly protected by maternal antibodies and the highest incidence rate is registered between 6 and 24 months of age, with the greatest risk of developing severe disease under age 12 months.^3,5^

Seasonality is a key feature of RV infections with peak incidence related to latitude and climate and, in most developed countries, a higher incidence of RVGE in cooler and drier seasons.^6,7^

RV infections are most certainly underreported, as RV is often not distinguished from other causes of AGE, even for patients hospitalized for diarrhea.^7^ The treatment of AGE is the same regardless of its cause, so the incentive to do the testing required for differential diagnostics is rather limited.^3^ The treatment of AGE is fluid replacement to prevent dehydration due to vomiting and diarrhea and zinc treatment, which may shorten the duration of diarrheal episodes.^1^ Since about 2006, 2 effective and safe vaccines against rotavirus have been available and recommended by WHO from 2007 but the uptake across the world has been highly variable.^1^ One reason for limited uptake in developed countries is uncertainty about the cost-effectiveness of the vaccines, partly because the knowledge about the actual burden of disease is limited.^8^

In Latvia, RVGE is a notifiable disease, but stool samples are not routinely tested for RV in primary care settings and most existing RVGE incidence estimates are based on hospital data. The RVGE incidence increased from 84 per 100,000 in 2007 to 169 per 100,000 in 2011.^9^ The highest incidence (20–30% above the national average) was observed in the regions around the two largest cities, Riga and Daugavpils.^4^ Latvia established national rotavirus vaccination recommendations in 2010 and fully reimbursed rotavirus vaccination was included in the National Immunization Program (NIP) from January 1, 2015. Within the first year of this full reimbursement, a coverage of 87% was achieved whereas coverage was < 5% when the vaccine was available in the private market from 2007.^10^

The objective of the present study conducted in Latvia was to investigate the burden of RVGE in primary care settings for infants and children under 5 years old prior to the introduction of RV vaccination in the NIP. We focused on estimating the proportion of RVGE among all AGE cases presenting in primary care, their severity as summarized by the Vesikari scale,^11^ the proportion of RVGE cases in primary care referred to hospital admission and the seasonality of RVGE episodes.

The data reported here may therefore serve as baseline data for future studies of the impact of vaccination on the disease burden and of the cost-effectiveness of vaccination.

## Results

### Demographic characteristics

Data were evaluable for 606 enrolled children (Table 1). Of these, 21 were enrolled more than once (and counted as separate cases), 7 of whom were RV-positive. The gender distribution was approximately equal overall and in each RV status group. The median age was a little higher in the RV-positive than in the RV-negative group, 24 months versus 20 months.

**Table 1.** Demographics characteristics of children included in the study by rotavirus (RV) status.

Subject group	RV-positive (N = 231)	RV-negative (N = 375)	Total (N = 606)
Gender (male/female) (%)	55.8/44.2	50.9/49.1	52.8/47.2
Age at GP pediatrician visit (months)			
Mean (SD)	26.5 (14.72)	23.52 (15.68)	24.66 (15.38)
Median (range)	24 (0–58)	20 (0–59)	21 (0–59)
Underlying medical condition; n(%)			
Prematurity	7 (3.0)	9 (2.4)	16 (2.6)
Pulmonary disease	5 (2.2)	6 (1.6)	11 (1.8)
Congenital disease	2 (0.9)	1 (0.3)	3 (0.5)
Gastrointestinal disease	2 (0.9)	1 (0.3)	3 (0.5)
Other	5 (2.2)	5 (1.3)	10 (1.7)
No condition	209 (90.5)	353 (94.1)	562 (92.7)

SD = Standard deviation

### Proportion of RVGE cases, overall and per age group

Among the 606 AGE cases, 231 (38.1%, 95% CI: 34.2–42.1%) were RV-positive. The proportion of RV-positive per age group (derived from Table 2), increased from 19.5% (9/46) in the infant group (0–5 months), to 42.8% (119/278) in the oldest group (24–59 months). The number of RVGE cases was similar in the group under 24 months old and in the group from 24 to 59 months, 112 and 119, respectively, but the proportion of RV-positive relative to all AGE cases was lower in the group under 24 months, 34.1% (112/328).

**Table 2.** Severity of acute gastroenteritis by rotavirus (RV) status and age group.

		RV-positive (N = 231)		RV-negative (N = 375)		
Age (months)	Severity (Vesikari Score)	n	%	n	%	p-values
0–5	Mild (1–6)	2	22.2	21	56.8	0.1510
	Moderate (7–10)	6	66.7	12	32.4	
	Severe (≥ 11)	1	11.1	4	10.8	
6–11	Mild (1–6)	4	16.0	27	42.2	0.0067
	Moderate (7–10)	9	36.0	26	40.6	
	Severe (≥ 11)	12	48.0	11	17.2	
12–23	Mild (1–6)	13	16.7	42	36.8	< 0.0001
	Moderate (7–10)	24	30.8	46	40.4	
	Severe (≥ 11)	41	52.6	26	22.8	
	Missing*	0	-	1	-	
24–59	Mild (1–6)	25	21.0	75	47.2	< 0.0001
	Moderate (7–10)	54	45.4	52	32.7	
	Severe (≥ 11)	40	33.6	32	20.1	
Overall	Mild (1–6)	44	19.0	165	44.1	< 0.0001
	Moderate (7–10)	93	40.3	136	36.4	
	Severe (≥ 11)	94	40.7	73	19.5	
	Missing*	0	-	1	-	

* Lost to follow-up; RV: Rotavirus; p-values = Result of Fisher’s exact test of the difference in severity according to RV status

### Clinical characteristics and severity

The severity of the AGE episode by age group and RV status is described in Table 2. Overall, 40.7% (94/231) RV-positive and 19.5% (73/375) RV-negative children experienced severe AGE (Vesikari score ≥ 11; p < 0.001). The same trend was seen within each of the separate age groups, with the statistical significance of the difference depending on the number of individuals in the age group. The most substantial differences were seen in the age groups 6–11 and 12–23 months, where about half the RV-positive and a fifth of the RV-negative children had experienced severe AGE, while there was no difference according to RV-status in the proportion of infants (0 to 5 months) with severe AGE. In the RV-negative patients, the proportions with severe AGE did not vary with the child’s age, but in the RV-positive patients there was a marked peak of severity in the group of 6 to 23 months old (similar in both subsets, from 6 to 11 and from 12 to 23 months).

Table 3 summarizes details of the symptoms experienced, on which the aggregate Vesikari severity score is based. All the symptoms were either more frequent, more severe or/and of longer duration in the RV-positive than in the RV-negative patients. The logistic regression models showed an association between dehydration (odds ratio (OR): 2.06, p < 0.001), fever (OR: 2.03, p < 0.001), vomiting (OR: 1.45, p = 0.048) and Vesikari score ≥ 11 (OR: 2.80; p < 0.001) and RV infection as the cause of AGE.

**Table 3.** Severity of symptoms by rotavirus (RV) status.

	RV-positive (N = 231)		RV-negative (N = 375)	
Symptom	n	%	n	%
**Diarrhea**				
Yes	227	98.3	370	98.9
No	4	1.7	4	1.1
Missing	0	-	1	-
Number of stools per day (maximum)				
1–3	20	8.8	51	13.8
4–5	104	45.8	195	52.7
≥ 6	103	45.4	124	33.5
Days of diarrhea				
1–4	139	61.2	258	69.7
5	44	19.4	41	11.1
≥ 6	44	19.4	71	19.2
**Vomiting**				
Yes	158	68.4	199	53.2
No	73	31.6	175	46.8
Missing	0	-	1	-
Number of vomiting episodes per day (maximum)				
1	20	12.7	52	26.1
2–4	102	64.6	115	57.8
≥ 5	36	22.8	32	16.1
Days of vomiting				
1	64	40.5	99	49.7
2–4	91	57.6	94	47.2
≥ 5	3	1.9	6	3.0
**Fever**				
Yes	182	78.8	227	60.7
No	49	21.2	147	39.3
Missing	0	-	1	-
Temperature				
37.1–38.4°C	40	22.0	80	35.2
38.5–38.9°C	65	35.7	71	31.3
≥ 39.0°C	77	42.3	76	33.5
**Dehydration**				
Degree of dehydration				
No dehydration	115	49.8	251	67.1
Mild/moderate (1–5%)**	103	44.6	116	31.0
Severe (≥ 6%)**	13	5.6	7	1.9
Missing	0	-	1	-
Type of treatment*				
Rehydration in polyclinic	50	21.6	40	10.7
Hospitalization	21	9.1	18	4.8

*: Type of treatment are not substitutes, each patient may have both,**: The percentages indicate the amount of weight loss.^21^

### Proportion of hospitalizations among AGE cases

Among the 606 children in the study, 39 were hospitalized. The rate of hospitalization was 9.1% (21/231) and 4.8% (18/375) for the RV-positive and RV-negative patients, respectively, with overlapping CIs (a borderline significant difference overall [p = 0.0485], see Table 4). The number of days in hospital was not significantly different according to RV-status, with a mean and median of 4.1 and 4 days, respectively, in the RV-positive group and 3.1 and 3 days, respectively, in the RV-negative group.

**Table 4.** Number of hospitalizations by rotavirus (RV) status and age group.

	RV-positive (N = 231)				RV-negative (N = 375)				
Age (months)	N	n	%	95% CI	N	n	%	95% CI	p-value
0–5	9	0	0.0	0.0–33.6	37	1	2.7	0.1–14.2	1.0
6–11	25	2	8.0	1.0–26.0	64	3	4.7	1.0–13.1	0.6173
12–23	78	10	12.8	6.3–22.3	115	10	8.7	4.2–15.4	0.6083
24–59	119	9	7.6	3.5–13.9	159	4	2.5	0.7–6.3	0.0813
All	231	21	9.1	5.7–13.6	375	18	4.8	2.9–7.5	0.0485

CI: confidence interval

A multivariate, stepwise logistic regression analysis of the determinants of hospitalization of the AGE cases was performed. Based on the results of univariate analyses of the risk factors for hospitalization, the saturated model included the following variables: Vesikari severity score (≥ 11 vs. < 11), gender, age (24–59 months, 12–23 months, 0–11 months) and RV-status (Table 5). In the multivariate analysis, only the Vesikari severity score was statistically significant (odds ratio = 21.34, p < 0.001), whereas age, gender and RV-status in itself had no independent impact on the risk when controlling for the other factors.

**Table 5.** Estimated odd ratios and p-values of the fitted logistic regression model for the possible risk factors for hospitalization of acute gastroenteritis patients.

Model	Risk Factor	p-value	OR	95% CI of OR
Saturated	Vesikari ≥ 11 vs < 11*	0 .0001	21.341	8.003–56.909
	Female vs male*	0.4313	0.753	0.371–1.527
	Age 12–23 vs 0–11 months*	0.2815	1.744	0.634–4.797
	Age 24–59 vs 0–11 months*	0.7840	0.863	0.302–2.467
	RV-positive vs RV-negative*	0.9711	0.987	0.481–2.023
Final	Vesikari ≥ 11 vs Vesikari < 11*	0 .0001	22.189	8.508–57.874

CI: confidence interval; OR; odds ratio; *reference category

### Seasonal distribution of RVGE

**Figure 1.**
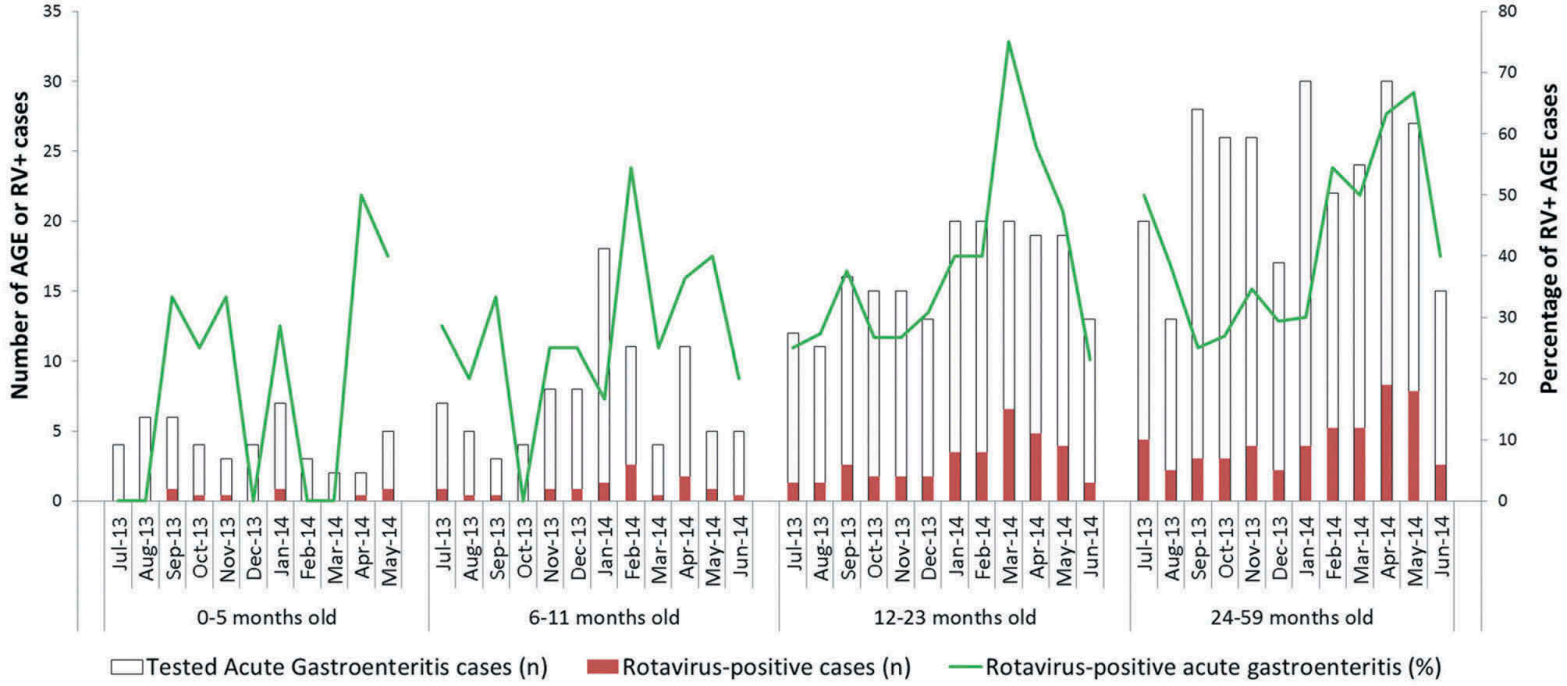
Seasonal distribution of acute gastroenteritis (AGE) cases and percentage of rotavirus-positive (RV+) cases by age group (months).

AGE and RVGE occurred throughout the year in this Latvian population of infants and young children but in each age group there was a marked seasonality in the proportion of RVGE cases relative to all AGE cases (Figure 1), which peaked in the months March to May 2014 with proportions of around 56% overall. The minimum proportion of RVGE relative to all AGE cases was observed in October 2013 with 24.5%. The absolute number of RVGE cases per month ranged from 9 in August 2013 to 35 in April 2014.

## Discussion

Until now, very limited information on the burden of RVGE in primary care settings in Latvia has been available. Our study underlines that RV is an important cause of AGE cases presenting in primary care in Latvia, causing the most severe forms of AGE disease and therefore requiring hospitalization more frequently than for AGE cases with other causes. For equally severe AGE cases, the RV status was not determinant for the risk of being admitted into hospital.

A companion study based on the same protocol was performed in Bulgaria with data collection during the calendar year 2013.^12^ The overall proportion of RV-positive cases was 25.5% in Bulgaria compared with 38.1% in Latvia but both studies found that the proportion of RV-positive cases increased linearly with age and more than doubled for children aged 24–59 months compared to the youngest infants aged 0–5 months. The severity assessments were very different in the two studies. In the Bulgarian study, the proportion of severe cases (Vesikari score ≥ 11) was 81.8% for the RV-positive and 54.6% for the RV-negative cases, compared with 40.7% and 19.5%, respectively, in the present study. In Bulgaria, the hospitalization rate was 20.1% for RV-positive and 1.5% for RV-negative cases, compared with 9.1% and 4.8%, respectively, in Latvia. This illustrates another finding in the Bulgarian study, namely that both the RV-status and the Vesikari score of the cases were independent risk factors for hospitalization, so even controlling for the severity of the case the child’s RV-status had a significant impact on the risk of hospitalization. By contrast, only a severe Vesikari score was found to be a significant risk factor for hospitalization in this Latvian study. Among the potential explanations of the divergent observations in the two countries are that the epidemiology of RV infections may be dissimilar and that healthcare at the local level may be organized differently; in Latvia, parents may go directly to the hospital to seek care for a sick child and this may suggest that the more severe cases of RVGE are seen less often in primary care.

Our observations are broadly similar to the findings of a number of other studies carried out in primary care settings in European countries.^13–19^ The REVEAL study^18^ which included 6 Western European countries reported an overall proportion of 53% with severe disease in RV-positive AGE patients. The ratio of RV-positive/RV-negative AGE patients with dehydration was about 1 in Belgium and the UK, but 1.82 in France, 5.54 in Germany, 3.27 in Italy, 3.47 in Spain and 2.18 in Sweden. The proportion of hospital referrals for RVGE varied from 13.0% (relative risk (RR) compared to non-RV AGE = 3.37) to 57.1% (RR = 2.10). Another noticeable difference from our findings is that the REVEAL study showed that 33–68% of children with AGE who first presented in a primary care setting subsequently required medical attention in another medical setting. We observed that 15% received rehydration in a polyclinic and that 6.4% were hospitalized. In the REVEAL study, up to 2/3 of all hospitalizations of AGE cases were due to RV ^18^ here it was 53.8% (21/39). It is difficult to assess whether these differences have any other meaning than random variation but there are clearly differences in treatment patterns between countries.

The proportion of RV-positive among all the AGE cases recorded peaked in the months March to May 2014 indicating seasonality of RV infections, a pattern which is not expected for AGE infections of other causes. This is in line with the conventional notion that seasonality of RV infections is characteristic of upper-middle and high income developed countries with a temperate climate.^20^ However, to reach a firm conclusion about the possible seasonality of RV infections in Latvia before widespread RV vaccination would require surveillance data for several years. Such analyses would also allow examination of the potential impact of RV vaccination on the seasonality of infections which has been observed in some countries, with delay of onset, shorter duration and dampening of peaks.^21^

It is clear that estimating the burden of RVGE by examining only AGE cases presenting in primary care does not give a complete picture of the burden of disease caused by RV, because most of the milder cases will be taken care of in the home without any medical attention. For Europe, a systematic review thus estimated that the proportion of RVGE cases receiving no medical attention varied between 25% and 51%.^22^

Our results must be interpreted with caution as the study was limited by a number of factors. The 52 study centers were located throughout Latvia with about 40% in the regions surrounding the largest cities where the RVGE incidence based on hospital data has been estimated to exceed the national average by some 20–30%. Apart from these data, we are not able to assess the representativeness of the primary care units selected and, as a consequence, the generalizability of the findings to the whole country is uncertain.

Another limitation of the study was that the severity rating was done by the investigator in an informal way during the follow-up phone call with the parents and based on the parents’ recall about the number, severity and duration of the episodes with symptoms. It is not possible to assess the accuracy of the parents’ recall but they had not received prior instructions to record each episode although they had been informed that they would be enquired about this. A scoring manual prepared for use of the Vesikari scale in clinical trials presupposes parent completion of daily diary cards and stringent control by specially trained study clinicians^23^ but adoption of such a strict protocol was not considered necessary for this study. The same informal severity scoring approach was used in the companion study in Bulgaria^12^ and we have no obvious explanation for the striking difference in the average severity scores observed in the two studies. As mentioned in the introduction, Latvia included fully reimbursed vaccination in the NIP in 2015. Observational impact studies in several industrialized countries have reported substantial reductions in disease burden occurring just a few years after implementation of RV vaccination, with some evidence also of herd immunity for unvaccinated older children and adults.^1,24^ Future epidemiological studies may show if the impact of increased coverage of RV vaccination in Latvia will be similar to that observed elsewhere despite the substantially increased RVGE incidence in the years prior to this change of the NIP. Such studies would require collection of data covering several years before and after widespread coverage of vaccination to enable a thorough analysis of the impacts of the vaccination strategy on the seasonal timing, geographical distribution and amplitude of RV infection in the susceptible populations. The present study with data collection over just one year in primary care is too limited to serve this purpose and must be seen as just a modest step in describing the epidemiology of RV in Latvia before general uptake of RV vaccination.

## Conclusion

This study of the proportion of AGE cases caused by RV infection in primary care in Latvia underlines that RV is an important cause of AGE and that RVGE more often is severe and leads to dehydration requiring treatment than AGE with other causes. Hospitalization for AGE-related symptoms was also more frequent RV-positive children.

## Materials and methods

### Study design

This prospective, active surveillance study (NCT01733849) was carried out with patient recruitment and data collection from July 2013 to the end of June 2014 in 52 primary care units throughout Latvia. The study was purely observational and no clinical management instructions were included. All clinical management including any decision about hospitalization was based exclusively on the clinical judgment of the pediatrician consulted without instructions or other influence from the study design.

Approximately 40% of the primary care units were located in the regions of the two largest cities (Riga and Daugavpils), the rest were located in rural areas. The number of units included was based on the targeted sample size and workload feasibility considerations, considering that each unit would be able to include 15–20 AGE cases over the 12-month recruitment period. Each primary care unit was instructed to enroll presenting eligible AGE patients as a function of the number of AGE cases in children under 5 years old it had reported during the preceding year. Thus, units which had had relatively few AGE cases the year before were instructed to seek to enroll each eligible AGE case presenting, whereas units which had treated relatively many AGE cases the year before were instructed to seek to enroll every second or third, etc. Children could be included more than once provided that they had been symptom-free at least 14 days since the end of the previous episode. With this provision, each repeat episode was recorded as a new case.

### Study population and case definition

Children younger than 5 years old, whose parents consulted their primary care physician for the child’s AGE, either in the clinic or as a home visit, were eligible for enrolment. A case of AGE was defined as 3 or more loose stools and/or 2 or more vomiting episodes within a 24-hour period with onset of symptoms ≤ 14 days before the consultation. There was no specific exclusion criterion and children were included if they met the definition of an AGE case and the parents gave informed consent. This study was conducted in accordance with Good Clinical Practice guidelines and the Declaration of Helsinki and was approved by the national independent ethics committee in Latvia. Written informed consent was collected from parents/guardians before enrolment.

### Data collection

During the consultation or home visit, details of the infant or child were recorded. The parents completed a questionnaire concerning their child’s age, gender, general medical history, AGE symptoms at presentation and date of onset of symptoms, previous RV vaccination, etc. The parents were asked to monitor GE symptoms and the condition and temperature of their child for the 13–18 days following enrolment. About 2 weeks after the consultation the investigator made a follow-up phone call to record the duration of diarrhea and vomiting and the maximum number of episodes of diarrhea and vomiting experienced per 24 hours during this observation period. The severity of the AGE case was assessed by means of the 20-point Vesikari scale, which is based on the intensity and duration of vomiting and diarrhea, intensity of fever and dehydration, and need for treatment and hospitalization (with the severity categories defined as: score ≤ 6: mild; score 7–10: moderate; score ≥ 11: severe).^11^ The severity scoring was performed by the investigator during the follow-up phone call for recording of symptoms.

### Laboratory analysis

A stool sample was collected from each child at the first visit or up to 4 days following this and was immediately tested for RV either at the clinic or during the home visit using a rapid visual immunochromatographic test kit with a sensitivity of 99.1% and specificity > 99.9% (*IMMUNOQUICK-RV*, Biosynex, France).^25^ The samples were not tested for other intestinal pathogens. In case the test could not be performed immediately after collection, the sample could be stored in a refrigerator for up to 24 hours at 2–8°C and tested when convenient.

### Statistical analysis

Studies in various European countries of children under the age of 5 years presenting in primary care with AGE have shown highly varying proportions of RVGE among these, ranging from 7.7% to 55%.^22,26^ Assuming an expected RVGE/AGE proportion of around 35% and an acceptable width of the 95% confidence interval (CI) of ≤10%-points, we considered that an evaluable sample of 700 patients would allow us to estimate the actual proportion of RVGE cases with sufficient precision, given the variability observed in the studies mentioned and given that the incidence of RV is known to vary substantially from year to year.^22^ Assuming that some 10% of the patients consenting to participate would be non-evaluable, we set the target sample size to 780.

The analysis was performed on all the enrolled patients, who complied with the procedures stipulated in the protocol and who had data available. The associations between disease symptoms and RV status and between hospitalization and RV status were analyzed by multivariate logistic regression modelling with Wald test of the significance of the coefficients. The variables included in these regression analyses comprised symptoms (present/absent), age, gender, Vesikari score, and RV-status. Differences with respect to categorical variables were tested by Chi-square tests and Student’s t-test was used for continuous variables (mean hospital days for admitted children). The statistical analyses were performed using SAS version 9.2.

## Disclosure of potential conflicts of interest

Dace Gardovska declares lecture fee from the GSK group of companies and Riga Stradiņš university research grant for investigation of RV serotypes in children in Latvia. Liga Kozlovska declares lecture fee received from the GSK group of companies. Kusuma Gopala and Monica Tafalla are employed by the GSK group of companies. Monica Tafalla holds shares in the GSK group of companies.
